# Effects of triptolide from *Radix Tripterygium wilfordii *(*Leigongteng*) on cartilage cytokines and transcription factor NF-κB: a study on induced arthritis in rats

**DOI:** 10.1186/1749-8546-4-13

**Published:** 2009-07-02

**Authors:** Cheng Xiao, Jing Zhou, Yinghui He, Hongwei Jia, Linhua Zhao, Ning Zhao, Aiping Lu

**Affiliations:** 1 Institute of Clinical Medicine Research, China-Japan Friendship Hospital, Beijing 100029, PR China; 2 Institute of Basic Theory, China Academy of Traditional Chinese Medicine, Beijing 100700, PR China; 3 National Pharmaceutical Engineering Centre for Herbal Preparations, Jiangxi University of Traditional Chinese Medicine, Nanchang, Jiangxi 330006, PR China; 4 E-institutes of Shanghai Municipal Education Commission (Shanghai University of Traditional Chinese Medicine), Shanghai 201203, PR China

## Abstract

**Background:**

Triptolide, an active compound of *Radix Tripterygium wilfordii*, is immunosuppressive, cartilage protective and anti-inflammatory both in human and animal studies of various inflammatory and autoimmune diseases, including rheumatoid arthritis, but its therapeutic mechanism remains unclear. The aim of this study is to investigate the effects of triptolide on cartilage cytokines in the CIA model.

**Methods:**

Sprague Dawley rats were immunized with type II collagen and orally administered with triptolide. The arthritic scores and incidence changes of the rats were observed. The expression of TNF-α, IL-6, COX-2 and NF-κB in paw cartilage was studied with immunohistochemical staining.

**Results:**

Triptolide, at both high and low doses, significantly lowered the arthritic scores, delayed the onset of arthritis and lowered the arthritis incidence. Triptolide treatment at both high and low doses lowered the expression of TNF-α, IL-6, COX-2 and NF-κB in paw cartilage in arthritic rats.

**Conclusion:**

Triptolide lowers the arthritic scores, delays the onset of collagen induced arthritis and reduces the expressions of TNF-α, IL-6, NF-κB and COX-2 in paw cartilage in arthritic rats.

## Background

Rheumatoid arthritis (RA) is a chronic disease that affects peripheral joints in human. It is characterized by inflammation and cellular proliferation in the synovial lining of joints, which can result in progressive destruction of cartilage and subchondral bones [1]. Tumor necrosis factor-α (TNF-α), interleukin-6 (IL-6), cycloxygenase-2 (COX-2) and nuclear factor κB (NF-κB) play important roles in the disease progression of RA [2-5].

Current treatments for RA include disease modifying antirheumatic drugs (DMARDs), non-steroidal anti-inflammatory drugs (NSAIDs), steroid and biological response modifiers which all aim to slow the disease progression and prevent further joint damage. Patients on these treatments may develop dependency on the medications and often suffer from side effects.

Triptolide is an active compound in the extract of *Radix Tripterygium wilfordii *(*Leigongteng*) [6,7]. Triptolide is immunosuppressive, cartilage protective and anti-inflammatory *in vivo *and effective on both humans and animals inflicted by a range of inflammatory and autoimmune diseases, such as RA [8-11]. The anti-inflammatory effects of triptolide include the inhibition of IL-2 production in mouse T cell hybridomas, inhibition of transcriptional activation of NF-κB, suppression of NF-κB in T lymphocytes [12] and reduction of PGE_2 _production in human monocytes and RA synovial fibroblasts [13]. An earlier study showed that triptolide reduced the incidence and severity of arthritis in collagen induced arthritis (CIA) model [14]. The effects of triptolide on cartilage TNF-α, IL-6, COX-2 and NF-κB are unclear; thus the present study investigates the effects of triptolide on cartilage cytokines in the rat CIA model, which is a widely used animal model of inflammatory polyarthritis with similarities to RA, and primarily mediated by an autoimmune response [15-18].

## Methods

### Animals

Sixty male Sprague Dawley rats of 8–10 weeks old (180–200 g body weight) were purchased from the Center for Laboratory Animal Care, Chinese Academy of Medical Sciences. Rats were randomly divided into four groups: (1) control, (2) CIA, (3) high dose triptolide (TH) and (4) low dose triptolide (TL). Rats were housed in a temperature-, humidity- and light-controlled environment with free access to rodent chow and water. The light-dark cycle was 12 hours (light phase from 06:00 to 18:00). The rodent license of the laboratory (NO. SYXK 11-00-0039) was issued by the Science and Technology Ministry of China. The approval of the Institutional Animal Ethics Committee was obtained before animal experiments were carried out.

### Induction of CIA and evaluation of arthritis

Soluble pure rat type II collagen (CII) was from Rikard Holmdahl (Lund University, Sweden) and complete Freund's adjuvant was purchased from Sigma Corporation, USA. CIA was induced by immunization once with 0.15 mg CII emulsified with equal complete Freund's adjuvant at the foot. Since eight days after immunization, the degree of arthritis was examined every two days. The severity of arthritis was expressed as mean arthritic index on a 0–4 scale according to the following criteria: 0- no edema, 1- slight edema and erythema limited to the foot and/or ankle, 2- slight edema and erythema from the ankle to the tarsal bone, 3- moderate edema and erythema from the ankle to the tarsal bone and 4- edema and erythema from the ankle to the entire leg. Each limb was graded and the maximum possible score was 16 for each animal. A score of one or above was considered arthritic.

### Treatment of CIA with triptolide

Triptolide (Huangshi Pharmaceutical Factory, China) was orally administered to the rats for one week after immunization, once a day, lasting for three weeks, at 18.62 μg per 5 ml per kg of body weight (TH) and 9.31 μg per 5 ml per kg of body weight (TL). The rats in the control and CIA groups were administered with 5 ml of saline per kg of body weight.

### Immunohistochemistry

The expression of TNF-α, IL-6, COX-2 and NF-κB in hind paw cartilage was determined with immunohistochemical staining kits (Wuhan Boster Biotech, China). The staining was performed in accordance with the product manual. Briefly, on day 28 after the immunization, hind paws were dissected, fixed in ice-cold 4% phosphate-buffered paraformaldehyde (pH7.4), decalcified in cold by 10% ethylenediamine tetraacetic acid (EDTA) in phosphate buffered saline (PBS) and cryostat-sectioned at 6 μm. The sections were fixed in cold acetone for ten minutes, washed in PBS and depleted of endogenous peroxidase by treatment with 0.3% H_2_O_2 _in absolute methanol for 15 minutes. After blocking nonspecific binding with 10% normal rabbit serum in PBS for 30 minutes, the sections were incubated with primary antibodies (rabbit anti-rat TNF-α, IL-6, COX-2 and NF-κB antibodies) at appropriate dilutions for one hour at 37°C, washed, incubated with biotinylated goat anti-rabbit immunoglobulin G (IgG,), washed and incubated with avidinbiotinylated horseradish peroxidase complex (ABC) and diaminobenzidine tetrahydrochloride DAB (Elite kit, Vector Laboratories, USA), then counterstained with hematoxylin. The positive granule averages of three samples (6 scoops in each sample) in each section were determined by Q-win DC100 Image Analysis (Leica Microsystems, Germany). The percentage of positive granules in the whole screened area was calculated.

### Statistical analysis

Student-Newman-Keuls test was employed for variable between groups when equal variances were assumed. Dunnetts's t test for equal variances not assumed. Chi-square test was employed for the incidence data analysis (SPSS version 11.0). *P *values less than 0.05 were considered statistically significant.

## Results

**Figure 1:**
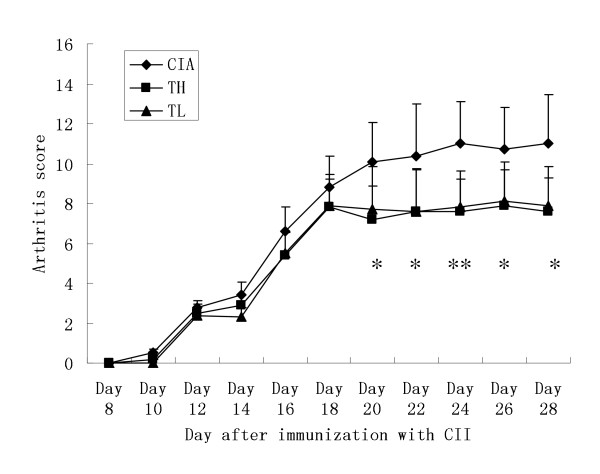
**Arthritic scores after immunization with CII**. Data are represented as mean ± standard deviation (SD). Since Day 20 after immunization, the arthritic scores decreased in the triptolide-treated rats at both high dosage and low dosage (*P < 0.01 and **P < 0.001 vs. CIA group).

**Figure 2:**
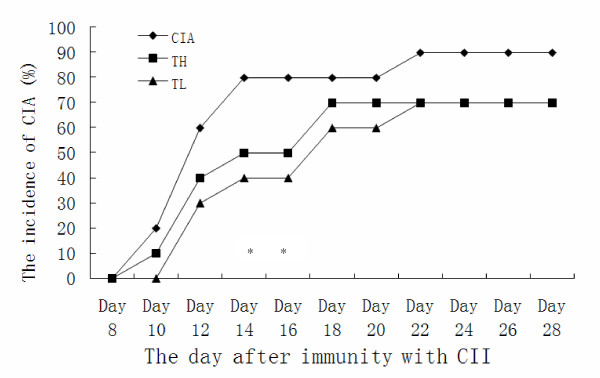
**Arthritic incidence of CIA in rats after immunization with CII**. Triptolide delayed the onset of arthritis (*P < 0.01 vs. CIA group).

Effects of triptolide on arthritic scores and incidenceWe used the CIA model in Sprague Dawley rats to investigate the effects of triptolide on arthritis. The changes of arthritis scores and arthritis incidence in the CIA and triptolide treatment groups are shown in Figures 1 and 2 respectively. CIA was developed in rats immunized with CII. Clinical signs (e.g. periarticular erythema and edema) of CIA first appeared in the paw on Day 10 after immunization (Figure 1). Day 20 after immunization, the arthritic scores started to decrease in the TH and TL groups (3.229<t<4.750, *P *< 0.01 or *P *< 0.001). Oral administration of TL to arthritic rats reversed paw edema as did TH (0<t<0.709, *P *> 0.05). Triptolide delayed the onset of arthritis (*P *< 0.01) and lowered arthritis incidence.

### Effects of triptolide on proinflammatory cytokines, COX-2 and NF-κB in cartilage

**Figure 3:**
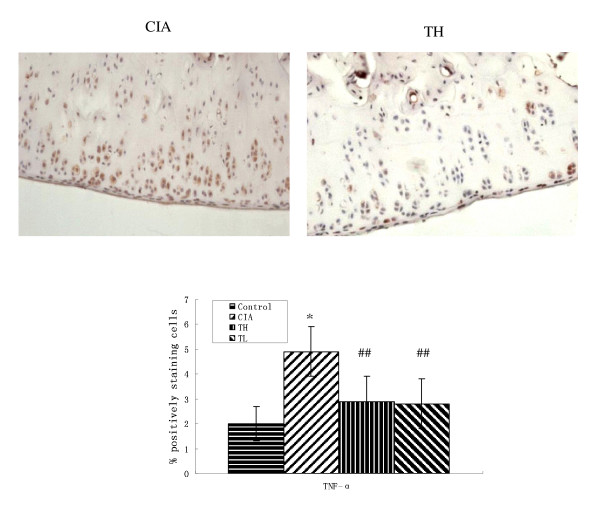
**Effects of triptolide on the expression of TNF-α in paw cartilage in the CIA group**. Paraffin sections of the joints were collected and subjected to immunohistochemical staining. TNF-α positive cells were stained in brown (hematoxylin counterstain). Data are represented as the mean ± SD (*P < 0.001 vs. control group; ##*P *< 0.01 vs. CIA group). (× 400).

**Figure 4:**
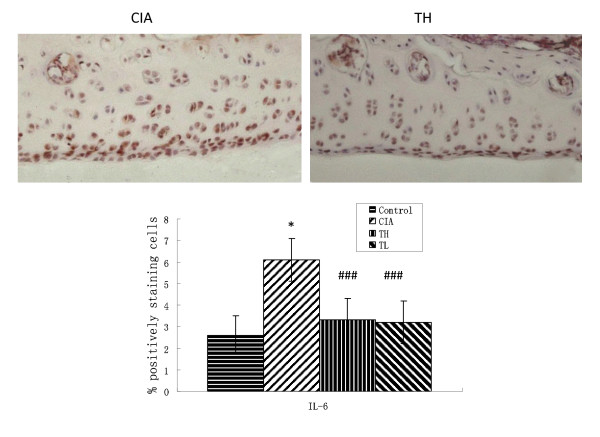
**Effects of triptolide on the expression of IL-6 in paw cartilage in the CIA group**. Paraffin sections of the joints were collected and subjected to immunohistochemical staining. The IL-6 positive cells were stained in brown (hematoxylin counterstain). Data are represented as the mean ± SD (*P < 0.001 vs. control group; ###*P *< 0.001 vs. CIA group). (× 400).

**Figure 5:**
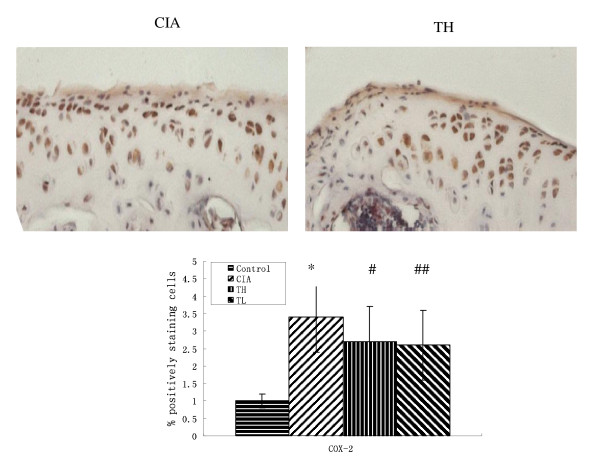
**Effects of triptolide on the expression of COX-2 in paw cartilage in the CIA group**. Paraffin sections of the joints were collected for immunohistochemical staining. The Cox-2 positive cells were stained in brown (hematoxylin counterstain). Data are represented as the mean ± SD (*P < 0.001 vs. control group; ##*P *< 0.05, #*P *< 0.01 vs. CIA group). (× 400).

**Figure 6:**
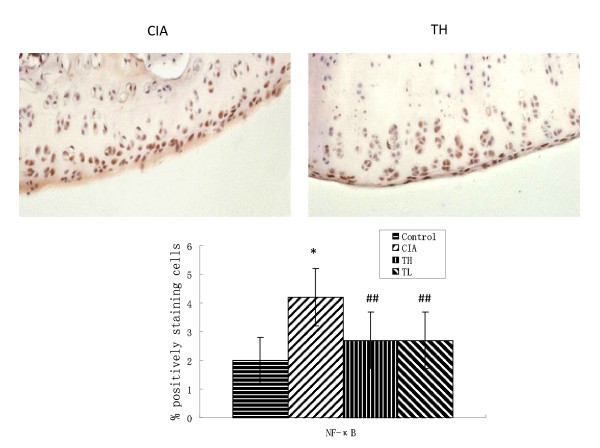
**Effects of triptolide on the expression of NF-κB in paw cartilage of CIA rats**. Paraffin sections of the joints were collected and subjected to immunohistochemical staining. The NF-κB positive cells were stained in brown (hematoxylin counterstain). Data are represented as the mean ± SD (*P < 0.001 vs. control group; ##*P *< 0.001 vs. CIA group). (× 400).

To investigate the actions of triptolide on CIA, we measured the levels of cartilage proinflammatory cytokines, COX-2 and NF-κB in joints. Immunohistochemical staining of paraffin sections of the joints femoral and tibial knee joint articular cartilage showed extensive positivity for TNF-α, IL-6, COX-2 and NF-κB (Figures 3, 4, 5 and 6). Positive cells markedly increased in the articular cartilage in the CIA group, more so in the superficial than deep layers, while positive immunostaining for TNF-α, IL-6, COX-2 and NF-κB in the control group was rare. In addition, articular cartilage treated with triptolide in both TH and TL groups showed limited and weak cytoplasmic staining for TNF-α, IL-6, COX-2 and NF-κB, which means their expression in superficial cartilage was lower than in that in the CIA group (TH vs. CIA: TNF-α t = 3.514, *P *= 0.0015; IL-6 t = 7.23, *P *< 0.001; COX-2 t = 3.152, *P *= 0.0038; NF-κB t = 4.536 *P *< 0.001; TL vs. CIA: TNF-α t = 3.41, *P *= 0.0019; IL-6 t = 6.618, *P *< 0.001; COX-2 t = 2.915, *P *= 0.0400; NF-κB t = 5.135 *P *< 0.001). The results showed that oral administration of TL to arthritic rats reduced the levels of cartilage cytokines TNF-α, IL-6, COX-2 and NF-κB as did TH (TNF-α t = 0.258, *P *= 0.7982; IL-6 t = 0.245, *P *= 0.8082; cox2 t = 0.411, *P *= 0.6841; NF-κB t = 0, *P *= 1.000).

## Discussion

RA is characterized by chronic inflammation in joints and destruction of cartilage and bone. Pro-inflammatory cytokines, such as TNF-α, IL-6, are closely associated with this pathologic process. At inflammatory sites of RA, TNF-α and IL-1 induce and/or enhance the production of prostaglandin E_2 _(PGE_2_) in synoviocytes and chondrocytes. IL-6 synergistically augments the inflammatory action of IL-1 in synoviocytes. The inflammatory actions of these cytokines are dependent on NF-kB and so is the expression of COX-2, the rate-limiting enzyme for PGE_2 _biosynthesis [2-5]. While previous studies focused on synovium and synoviocytes, the studies on the expression of TNF-α, IL-6, COX-2 and NF-κB in cartilage are lacking. The present study focuses on the pro-inflammatory actions of cytokines, i.e. the expression of TNF-α, IL-6 and NF-kB's in cartilage, in a CIA rat model. Immunohistochemical staining of the articular cartilage of the femoral and tibial joint showed that the expression of TNF-α, IL-6, COX-2 and NF-κB markedly increased in the articular cartilage, especially in its superficial layers. The present study shows that articular cartilage and chondrocytes play important roles in CIA.

*Radix Tripterygium wilfordii*, which demonstrated efficacy on inflammatory and autoimmune diseases such as RA, is immunosuppressive, cartilage protective and anti-inflammatory *in vivo *[19-24]. Triptolide is an active compound of *Radix Tripterygium wilfordii *and the anti-inflammatory effects of triptolide are achieved through the inhibition of NO production and iNOS expression through blockade of NF-κB and Jun N-terminal kinases (JNK) activation [16,25], inhibition of the proliferation of lymphocytes [17], down-regulation of TNF-α-induced COX-2 and production of PGE_2 _[18]. In the present study, we demonstrated that triptolide, at both high dose and low dose, significantly lowered the arthritic scores, delayed the onset of arthritis and lowered the arthritis incidence. Triptolide, at both high dose and low dose, lowered the expression of TNF-α, IL-6, COX-2 and NF-κB in cartilage in the CIA group. Triptolide, therefore, may have the potential to protect cartilage in patients of RA.

## Conclusion

Triptolide lowers the arthritic scores, delays the onset of CIA and reduces the expressions of TNF-α, IL-6, NF-κB and COX-2 in paw cartilage in rats.

## Abbreviations

ABC: avidinbiotinylated horseradish peroxidase complex; CII: type II collagen; CIA: collagen induced arthritis; COX-2: cyclooxygenase-2; DAB: diaminobenzidine tetrahydrochloride; DMARDs: disease modifying antirheumatic drugs; EDTA: ethylenediamine tetraacetic acid; ICAM-1: intercellular adhesion molecule-1; IFN: Interferon; IgG: immunoglobulin G; IL: interleukin; iNOS: inducible nitric oxide synthase; MMP: matrix metalloproteinases; JNK: Jun N-terminal kinases; NFκB: transcription factor κB; NO: nitric oxide; NSAIDs: non-steroidal anti-inflammatory drugs; RA: rheumatoid arthritis; PBS: phosphate buffer; PGE_2_: prostaglandin E_2_; SD: standard deviation; TNF: tumor necrosis factor.

## Competing interests

The authors declare that they have no competing interests.

## Authors' contributions

CX conducted the experiments, interpreted the results and prepared the manuscript. JZ designed the study, interpreted the results and prepared the manuscript. YHH designed the study and interpreted the results. HWJ interpreted the results. LHZ prepared the manuscript. NZ interpreted the results and prepared the manuscript. APL designed the study and revised the manuscript. All authors read and approved the final version of the manuscript.
